# A Comparative In Vivo Study of Tissue Reactions to Four Suturing Materials

**Published:** 2010-05-20

**Authors:** Shahla Kakoei, Fahimeh Baghaei, Shahriar Dabiri, Masoud Parirokh, Sina Kakooei

**Affiliations:** 1. Department of Oral Medicine, Oral and Dental Diseases Research Center, Dental School, Kerman University of Medical Sciences, Kerman, Iran.; 2. Department of Pathology, Oral Pathology Department, Dental School, Hamadan University of Medical Sciences, Hamadan, Iran.; 3. Department of Pathology, School of Medicine, Kerman University of Medical Sciences, Kerman, Iran.; 4. Department of Endodontics, Oral and Dental Diseases Research Center, Dental School, Kerman University of Medical Sciences, Kerman, and Iranian Center for Endodontic Research, Tehran, Iran.; 5. Department of Veterinary Medicine, Veterinary School, Shahid Bahounar University, Kerman, Iran.

**Keywords:** Catgut, Histopathologic, Oral, Polyglycolic Acid, PVDF, Silk, Sutures

## Abstract

**INTRODUCTION:**

The purpose of this study was to compare the histopathologic reaction of four suturing materials: silk, polyvinylidene fluoride (PVDF), polyglycolic acid, and catgut in the oral mucosa of albino rabbits.

**MATERIALS AND METHODS:**

The twenty-one male mature albino rabbits which were used in this study were randomly divided into three groups of seven each. Silk, PVDF, polyglycolic acid and catgut suture materials were tested in the oral mucosa of these animals. The animals were sacrificed 2, 4, and 7 days after suturing. Two pathologists evaluated the samples by determining the presence and level of inflammation, granulation tissue, and fibrosis formation. Data were statistically analyzed by Kruskal Wallis and Mann-Whitney U tests.

**RESULTS:**

Histological features of the samples showed that PVDF and plain catgut suture materials produced more fibrous tissue (favorable response) on the fourth day in comparison with silk suture (P=0.02). Also, in the 7-day samples PVDF sutures produced the mildest inflammation when compared with the silk sutures (P=0.015).

**CONCLUSION:**

According to the results of this study, it can be convey that PVDF suture materials created mild tissue reactions and can be a reasonable candidate for suturing oral tissues.

## INTRODUCTION

The use of appropriate suturing material and technique assists wound closure in general, oral and endodontic surgery. The aim of wound closure is to assist efficient healing and the return to function, as well as maintain the esthetics of the surgical site [1].

Research has shown that reduced accumulation of inflammatory cells around suture materials will accelerate wound healing [2]. Tissue reaction to suture materials is particularly important in patients who are susceptible to infection e.g. diabetic patients or patients taking immunosuppressive drugs [3].

The properties of an ideal suturing material includes ease of handling and knot tying, biocompatibility, and presence of smooth surface to prevent bacterial growth and wicking effect of oral fluids [4][5][6].

There are limited reports on the oral tissue reactions to suture materials [7][8][9]. One of the main reasons for the frequent application of silk sutures is due to the lack of research studies on alternative or new suture materials. Most experiments investigating tissue reactions to suture materials have been performed on the skin [7][8][9][10]. However, the epidermis does not emulate the oral cavity environment; i.e warmer temperatures, continuous intake of. food (change in pH and mechanical forces), various types of microbial flora, and the moist environment. Therefore, tissue reaction to suture material in the skin could be considerably different [11]. Recent research has highlighted the incomplete and inconsistent reports evaluating tissue reactions to different suture and closure materials [7][8][9].

Although some studies have reported that silk suture materials produced a more intense and prolonged inflammatory reaction in gingival and oral mucosa [2][7], it is still the most popular suture material used by dentists [4]. The braided and nonabsorbable quality and the tissue wicking effect that encourages plaque accumulation can cause severe inflammation in the incision site [11].

Polyglycolic acid suture material is a braided absorbable synthetic suture material [12], Catgut on the other hand, is an absorbable suture material frequently used in oral surgery [6]. Polyvinylidene fluoride (PVDF) is a monofilament suture material which has been successfully used in vascular surgeries [13][14][15][16]. Parirokh et al., in a scanning electron microscope (SEM) study, showed that contamination of PVDF suture material was significantly less than silk sutures in the oral mucosa of rabbits [17].

There is no histopathologic study that analyzes PVDF suture materials in the oral mucosa. Therefore, the aim of this study was to compare these four different suture materials in rabbit oral mucosa.

## MATERIALS AND METHODS

The research protocol was approved by the Research Ethics Committee of Kerman University of Medical Sciences (No. KA/85/45). In this experimental study, twenty-one adult male albino rabbits weighting 2.5-3 kg were used. All animals were subjected to an intra-peritoneal injection with 7.5 mg/kg Ketamine HCl (Alfasan, Woerden, the Netherlands) and 0.1 mg/kg Xylazine (Alfasan, Woerden, the Netherlands). After anesthesia, the head and neck area of the animals were scrubbed with betadine (Povidone-iodine, Daroupakhsh, Tehran, Iran) and their mouths rinsed with chlorhexidine gluconate 0.2% (Sharedaru, Tehran, Iran) mouthwash. Infiltration injection with lidocaine 2% with 1:80000 epinephrine (Daroupakhsh, Tehran, Iran) was then made posterior to the suturing site of the maxilla and mandible of each rabbit. The four different types of size 4.0 suture materials, silk (Supa, Tehran, Iran), polyglycolic acid (CG absorb, Supa Tehran, Iran), plain Catgut (Supa, Tehran, Iran), and PVDF (CG, Tehran, Iran) were applied in the buccal mucosa of the maxilla and mandible. The animals received soft diet till the end of the experiment.

The rabbits were randomly divided into three experimental groups (day 2, 4 and 7). After days 2, 4, and 7, the animal in each group were sacrificed and the suture placement areas were removed in block section. The tissues were kept in formalin 10% for 14 days. After tissue processing and H&E staining, the specimens were observed by two blinded pathologists. The pathologists were calibrated before specimen evaluation. Where disagreement occurred, the specimen was reevaluated and discussed by both pathologists to reach a definitive conclusion.

The tissue reactions immediately adjacent the sutures were assessed [11]. The specimens were evaluated for intensity of inflammation, epithelial proliferation, granulation tissue, and fibrosis formation. The evaluation criteria are outlined below.

***Intensity of inflammation:***

Presence of inflammatory cells at three different microscopic fields with ×1000 magnification around the suture material:

0 Absence of inflammatory cells

1 Mild infiltration of inflammatory cells (≤25)

2 Moderate infiltrations of inflammatory cells (50)

3 Dense infiltrations of inflammatory cells (≥75)

***Epithelial Proliferation:***

0 Absence of epithelial proliferation

1 Presence of epithelial proliferation

***Granulation tissue formation:***

0 Absence of granulation tissue

1 Presence of granulation tissue

***Fibrosis formation:***

0 Absence of fibrosis formation

1 Presence of fibrosis formation

The data were analyzed by Kruskal Wallis, and Mann-Whitney U tests. Bonferroni correction was used for pair-wise comparisons.

## RESULTS

There was no significant difference in epithelial proliferation, granulation and fibrous tissue formation between the suture materials (P>0.05). Overall, silk suture materials showed significantly more inflammation than catgut suture materials irrespective of the time interval (P=0.003).

At the two-day interval, histological observation showed no significant difference between suture materials for all the criteria (Table 1). Inflammatory cells, mostly macrophages and polymorphonuclear (PMN), were predominant around the suture materials.

**Table 1:** Mean ranks of histopathological tissue reaction in different time intervals

**Time intervals **	**2 days**			**4 days**			**7 days**		
****	***IF a***	***GT b***	***FF c***	***IF***	***GT***	***FF***	***IF***	***GT***	***FF***
**Silk**	11.75	11	11.75	16.07	10.5	7.36	13.33	9.17	7.5
**PVDF**	9.5	10.67	11.75	12.17	15.25	17.17	6.21	10.5	11.5
**PGA**	15.5	11.5	10	13.7	11.6	9.8	7.38	6.13	6.88
**catgut**	8.3	11	10	7.67	12.83	16.08	-	-	-
***P value d***	0.22	0.99	0.66	0.14	0.61	0.02	0.015	0.11	0.09

^a^ Inflammation

^b^ Granulation tissue

^c^ Fibrous formation

^d^ Kurskal Wallis test

**Figure 1:**
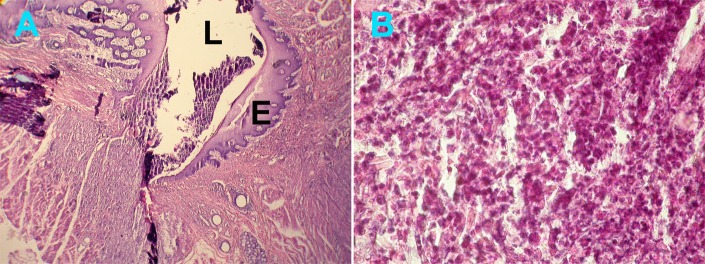
A) Epithelial proliferation around silk suture material in the 4-day specimen (×5), B) Inflammatory cells in the supporting tissue (×10) (L=Lumen, E= Epithelium)

**Figure 2:**
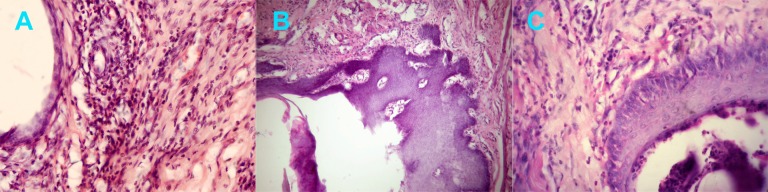
Dispersed inflammatory cells in the supporting tissues of A) PVDF suture material of the 4-day interval (×40), B) Catgut suture material in the 4-day specimens (×10), and C) PGA suture material (×40).

In the four-day samples, more fibrous tissues were observed around PVDF and plain catgut sutures than silk sutures (P=0.02) (Figure 2). Overall, for all groups, inflammatory cells were less compared to the previous interval. Microscopic evaluation showed dispersed inflammatory cell around and sometime inside the insertion site lumen of suture materials. Some, exhibited epithelial proliferation around the lumen (Figure 1A and Figure 1B).

Silk sutures had significantly greater inflammatory reaction on the 7th day compared to PVDF sutures (P=0.015). Also, almost all plain catgut and some of polyglycolic acid suture materials were reabsorbed (Table 1). PVDF sutures showed greater fibrous tissue formation in comparison with silk and the remnant polyglycolic acid sutures;this was statistically significant (P=0.09).

## DISCUSSION

The results of this study illustrated the diverse tissue reactions to test suture materials. There are some controversies surrounding the reasonable post-operative time for suture removal. Two till seven days have been suggested for this purpose [1][4][5][6][18]. Endodontic references suggest that anything between 2-7 days after periapical surgery would be reasonable for removing sutures [4][5][6]. However, oral and periodontal surgery references have suggested that 5-7 days could be ideal for suture removal [1][18]. In this study, different time intervals were employed to gauge which could be most appropriate.

Selvig et al. believe that delaying the removal of sutures post-operatively may increase the chance of bacterial contamination at the surgical site [11]. Banche et al. have recently expressed concern pertaining to the bacterial contamination of sutures; they have concluded that sutures should be removed as soon as practically possible [19]. In this study, silk sutures showed significantly more inflammatory reaction in comparison with PVDF suture material in the 7-day interval samples (P=0.015). A whole host of previous studies have shown similar results when silk sutures were compared with other suture materials [2][11]. A recent published SEM study showed that the braided configuration of the silk sutures (by wicking effect) encouraged microbial contamination of the whole surface just 3 days after suturing. However, PVDF sutures showed only 6.4% contamination of their surfaces after the same period [17]. Therefore, in the present study significant difference in inflammatory reaction between silk and PVDF suture materials may be attributed to the different degree of bacterial colonization over the materials.

Presence of fibrous tissue is a sign of regeneration [7][11]. At the 4-day interval, the PVDF and plain catgut samples had significantly greater fibrous tissue formation in comparison to silk sutures specimens. Based on previously published studies, the wicking effect in silk sutures occurred following their application in oral environment [5][17] and therefore, superior regeneration in the PVDF and plain catgut specimens may be due to reduced bacteria accumulation over these suture materials. In this study, all catgut and most polyglycolic acid suture (resorbable) materials disappeared by the 7-day interval. Previous studies also showed complete absorption by 7 days. [7][11][12][13], though one recent study reported the presence of polyglycolic acid suture materials eight days after suturing [20]. Many references do not recommend absorbable suture materials due to the variability in their rate of resorption; sutures may weaken and dissolve early or remain in the incision area for too long [5][6]. Absorbable suture materials incite varying degrees of tissue response due to degradation by hydrolysis, enzymatic digestion or phagocytosis [21].

In this study, the two-day interval showed no significant change in tissue reaction among all types of suture materials. Selvig et al. suggested that the acute response early on (1-2 days) may be attributed to suturing trauma, which is similar for all materials [11].

One study revealed that synthetic monofilament suture materials produce less tissue reactions. A SEM study on PVDF suture material showed that even in the 7-day samples, < 50 percent of the sutures’ surface area showed plaque contamination [17]. In this study PVDF (monofilament suture) material showed minimum tissue reactions at the 7 day interval.

There are a range of studies that compare the reaction of the body skin to suture materials [7][8][9]. Yaltirik et al. evaluated inflammatory reaction to silk, vicryl, polypropylene, and catgut on the dorsal portion of rat’s skin [7]. They found that vicryl suture materials produced milder inflammatory reactions compared to the three other suture materials. The key difference is that in oral tissue the suture material is immersed in saliva. Saliva contains an abundant supply of bacterial species that can penetrate underling tissue through the suture materials [5]. Therefore, for conclusive results, evaluation of various suture materials should be performed in the oral mucosa, irrespective of previous reports.

A suture material that remains longer than the desired time would increase the chance of underlying tissue contamination and interfere with tissue healing by inducing inflammation [3][11].

## CONCLUSION

Overall, PVDF suture material and catgut suture materials produced a milder tissue reactions compared with the other suture materials. This study illustrated the advantages of monofilament (PVDF) sutures.

These suture materials can be a reasonable candidate for suturing of oral tissues after surgery, particularly when longer times are required. Moreover, microscopic evaluation of the suture materials can be an effective method to compare inflammation and fibrous connective formation.
